# Macroalgal Morphogenesis Induced by Waterborne Compounds and Bacteria in Coastal Seawater

**DOI:** 10.1371/journal.pone.0146307

**Published:** 2016-01-08

**Authors:** Jan Grueneberg, Aschwin H. Engelen, Rodrigo Costa, Thomas Wichard

**Affiliations:** 1 Institute for Inorganic and Analytical Chemistry, Jena School for Microbial Communication, Friedrich Schiller University Jena, Jena, Germany; 2 Center of Marine Sciences, University of Algarve, Faro, Portugal; University of New South Wales, AUSTRALIA

## Abstract

Axenic gametes of the marine green macroalga *Ulva mutabilis* Føyn (Ria Formosa, locus typicus) exhibit abnormal development into slow-growing callus-like colonies with aberrant cell walls. Under laboratory conditions, it was previously demonstrated that all defects in growth and thallus development can be completely abolished when axenic gametes are inoculated with a combination of two specific bacterial strains originally identified as *Roseobacter* sp. strain MS2 and *Cytophaga* sp. strain MS6. These bacteria release diffusible morphogenetic compounds (= morphogens), which act similar to cytokinin and auxin. To investigate the ecological relevance of the waterborne bacterial morphogens, seawater samples were collected in the Ria Formosa lagoon (Algarve, Southern Portugal) at 20 sampling sites and tidal pools to assess their morphogenetic effects on the axenic gametes of *U*. *mutabilis*. Specifically the survey revealed that sterile-filtered seawater samples can completely recover growth and morphogenesis of *U*. *mutabilis* under axenic conditions. Morphogenetic activities of free-living and epiphytic bacteria isolated from the locally very abundant *Ulva* species (i.e., *U*. *rigida*) were screened using a multiwell-based testing system. The most represented genera isolated from *U*. *rigida* were *Alteromonas*, *Pseudoalteromonas* and *Sulfitobacter* followed by *Psychrobacter* and *Polaribacter*. Several naturally occurring bacterial species could emulate MS2 activity (= induction of cell divisions) regardless of taxonomic affiliation, whereas the MS6 activity (= induction of cell differentiation and cell wall formation) was species-specific and is probably a feature of difficult-to-culture bacteria. Interestingly, isolated bacteroidetes such as *Algoriphagus* sp. and *Polaribacter* sp. could individually trigger complete *Ulva* morphogenesis and thus provide a novel mode of action for bacterial-induced algal development. This study also highlights that the accumulation of algal growth factors in a shallow water body separated from the open ocean by barrier islands might have strong implications to, for example, the wide usage of natural coastal seawater in algal (land based) aquacultures of *Ulva*.

## Introduction

**Fig 1:**
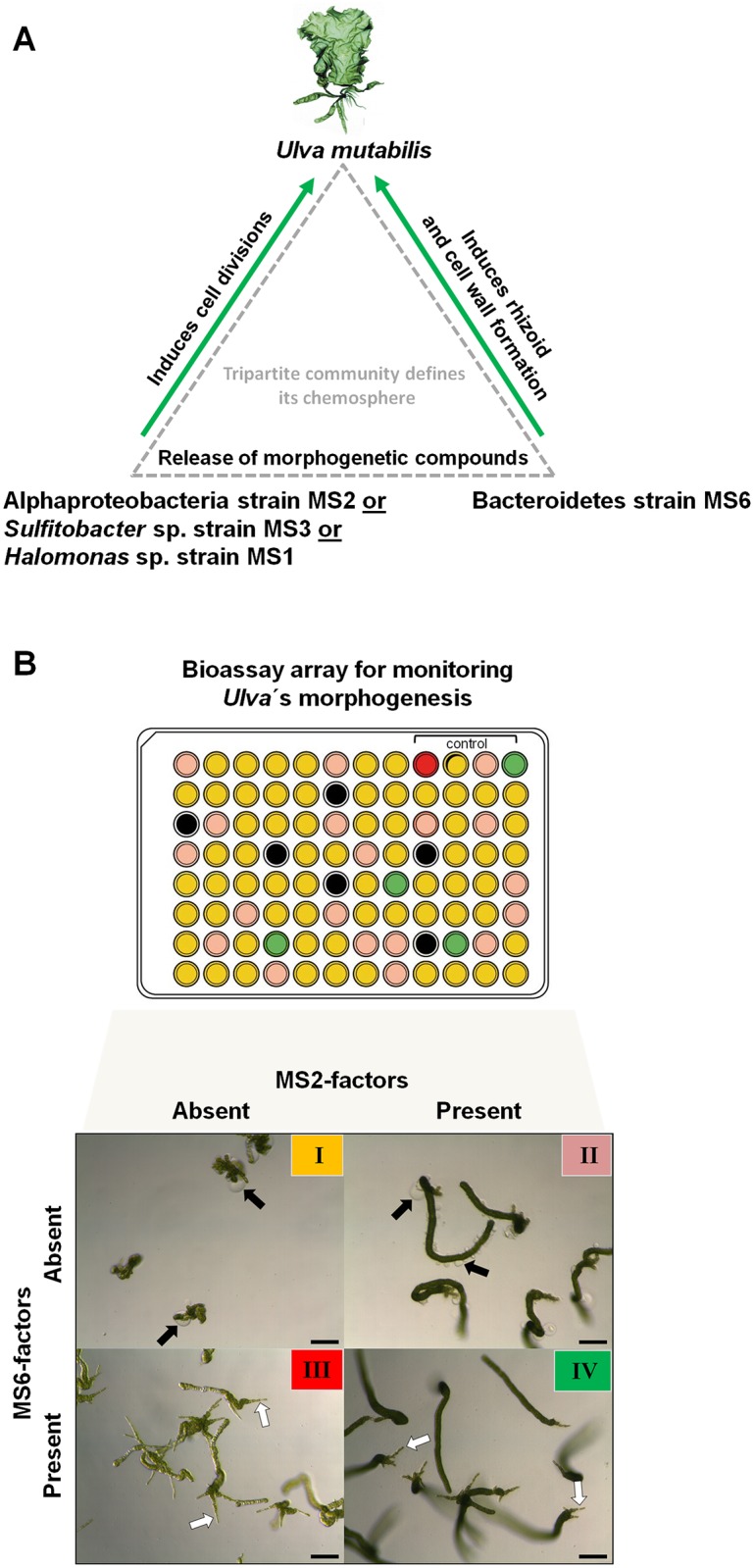
Categorization of the algal morphology of *U*. *mutabilis*. **(A)** The tripartite community of *U*. *mutabilis* with the essential interactions investigated in this study using a standardized experimental set-up. One of the strains MS1, MS2 or MS3 along with MS6 recovers completely growth, development and morphogenesis of *U*. *mutabilis*: Strains MS1 (*Halomonas* sp.), MS2 (originally classified as *Roseobacter* sp.) and MS3 (*Sulfitobacter* sp.) induce cell division and growth, whereas MS6 (originally classified as *Cytophaga* sp.) promotes rhizoid and cell wall formation [11]. **(B)** “*Ulva* bioassay array” for morphogenesis assessment. The multiwell based survey of the morphogenetic activity using axenic gametes of *U*. *mutabilis* allows the fast determination of the various morphotypes categorized by a color code. Representative morphotypes **I:** callus like morphology of an axenic culture (yellow); **II:** cell divisions and growth of blade cells with malformed cell walls (black arrow) in the presence of only MS2 (pink); **III:** Rhizoid formation (white arrow) and normal cell wall formation in the presence of only MS6 (red) **IV:** Complete morphogenesis in the presence of both strains MS2 and MS6 (green). Images (I-IV) were taken from Wichard (2015) *Front*. *Plant Sci*. 6:86 (black bar = 100 μm).

The sea lettuce *Ulva*, a green cosmopolitan macroalga, is known for its ability to form massive blooms called green tides, particularly in nutrient-rich coastal waters. Such events, which occur regularly in some regions, fill shores with tons of algal material [1–3]. While green tides have often been attributed to eutrophic water pollution [1,4–7], algal growth in the laboratory also depends on the external delivery of growth promoting morphogenetic compounds from bacteria [8–11]. Cultivation attempts of *Ulva* failed under axenic conditions in defined artificial seawater containing only essential metals, nutrients and vitamins. Moreover, Provasoli and co-workers (1958) added a soil extract into the culture medium to keep the algae growing properly [8,12]. Decades later, studies proved the impact of associated bacteria on the induction of algal growth, development and morphogenesis [11,13–19]. Although most discovered bacteria induced algal growth to a certain extent, they were not able to induce complete algal morphology solely. Spoerner et al. (2012) described then a tripartite symbiosis for *Ulva mutabilis* where bacterial strains MS2 (originally classified as *Roseobacter* sp.) and MS6 (originally classified as *Cytophaga* sp.) recover synergistically the complete morphogenesis (Fig 1A) [11]. In fact, a strictly axenic culture of *U*. *mutabilis* derived from purified gametes develops into a callus-like morphotype without the development of a holdfast with only scarce growth by cell division, as well as the occurrence of cell wall distortions (Fig 1B, black arrows). MS2, among other bacteria isolated from *U*. *mutabilis* such as *Sulfitobacter* sp. strain MS3 or *Halomonas* sp. strain MS1, induces cell division and growth of algal blade cells in co-cultivation experiments with axenic *Ulva* gametes. These germlings however were completely covered with the same bubble-like structures as in axenic *Ulva* cultures. Rhizoid growth did not occur either and cell wall distortions remained until either the strain MS1, MS2 or MS3 were also inoculated with strain MS6, leading to the formation of a normal cell wall and rhizoid (Fig 1B, white arrows). For instance, the combination of strains MS2 and MS6 revealed a full normal growth and morphology of *U*. *mutabilis* where the bacteria also grow and form a biofilm close to the rhizoid (Fig 1). This specific tripartite community became thus an ideal model and reference system that allows us to achieve controlled repeatable conditions in order to study, for example, the bacteria induced algal morphogenesis [20].

*U*. *mutabilis* was originally collected by Føyn in the Ria Formosa lagoon in southern Portugal in 1952 [21,22]. This new species was named “*Ulva mutabilis*”, because among the offspring, a wide variety of different developmental mutants spontaneously arose at an unusually high frequency due to genetic instability [21–24]. Among those mutants, one specifically stood out with a faster growth rate. Since then, many experiments have been performed using this ribbon-shaped mutant called “slender”, as opposed to the typical lettuce-like shape of the wildtype. However, the MS2 and MS6 strains together always induce the predisposed morphotype such as either the wildtype or slender, respectively [11]. Since 1952, these algae have been kept in the laboratory and bacteria have finally been isolated from the surface of lab-domesticated *U*. *mutabilis* by Spoerner et al. (2012).

In contrast to certain earlier studies, which stated the necessity of physical cell-cell contact between bacteria and alga [15], Spoerner et al. (2012) demonstrated that the bacterial-algal cross-talk even occurs in a two-chamber system when bacteria and axenic gametes are separated by a semi-permeable membrane. Thus, *U*. *mutabilis* perceives morphogenetically active and diffusible compounds from the medium [11].

Several studies have already highlighted that interactions between macroalgae and bacteria depend strongly on positive and negative chemical stimuli [25–28]. These chemical cues are often called infochemicals, and their identification is thus essential to the understanding of signal-mediated cross-kingdom interactions [17]. Algal growth-stimulating substances may be released by epiphytic bacteria in excess in the environment [17] and distributed *via* tidally driven transport mechanisms [29]. Land drainage favors further microbial growth and might trigger the production of morphogenetic regulators essential for the growth of seaweeds [8].

**Fig 2:**
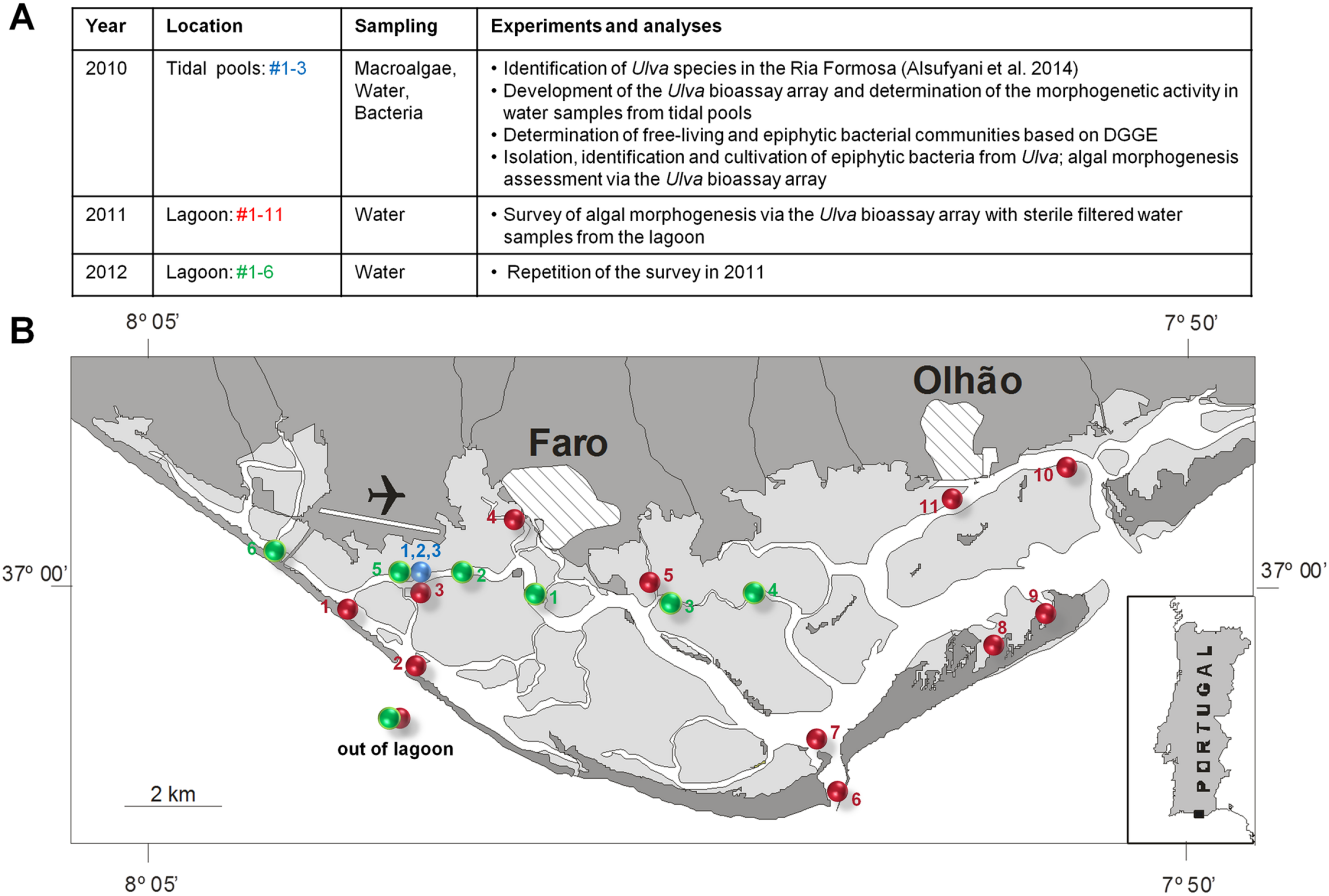
Ria formosa lagoon. (**A**) Scheme and purpose of sampling. (**B**) The morphogenetic activity of sterile-filtered water collected from several sampling sites within the lagoon [(1–3: Ramalhete Marine Station, blue dot) in 2010, (1–11, red dots) in 2011 and (1–6, green dots) in 2012] and two control samples outside the lagoon was tested on axenic *Ulva mutabilis* gametes sl mt(+). The numbers of the sites represent the chronological order of the sampling.

In this study, the sampling area, Parque Natural da Ria Formosa, is surrounded by barrier islands that enable water influx at three distinct places and consists of water-bearing main channels allowing boats and small ships to access the harbors in the urban regions of Faro and Olhão (Fig 2). Groundwater and nitrogen discharge may be important for triggering winter algae blooms on the mudflats, with little access to ocean water, as well as for the summer algae, which seemingly depend on the Ria Formosa as a nitrogen source [30]. The main parts of the lagoon are salt marshes and mudflats which are exposed at low tide, creating sandy flats or tidal pools, but are completely submerged at high tide. It was hypothesized earlier by Provasoli et al. (1958) that besides nutrients, fluctuations in the concentration of morphogenetic compounds could control the speed and size of green tides in coastal zones. However, the importance of morphogenesis in relation to their role in terms of ecosystem structure and function is still unknown [17]. To resolve these issues, not only are extensive pure culture studies needed, but explorative surveys for assaying plant hormones in seawater are also required [8]. This study thus aimed to determine the waterborne morphogenetic activities in lagoon water and in tidal pools inhabited by *Ulva* spp. which might function as natural sinks for bacterial morphogenetic substances. Initially, a robust and fast bioassay for algal morphogenesis was developed using the fast growing developmental mutant slender of *U*. *mutabilis* to survey reliably a large number of samples covering the area of the lagoon. While assessing the role of waterborne morphogenetic compounds for the green macroalga *Ulva* in the Ria Formosa, we determined the morphogenetic activity in sterile-filtered lagoon water collected during three spring seasons, as well as the bacterial communities in the lagoon water and on *Ulva*´s surface by denaturing gradient gel electrophoresis (DGGE). To verify the hypothesis of functional redundancy of various bacteria, as previously suggested [11], epiphytic bacteria were isolated and applied for morphogenesis-induction bioassays under standardized conditions (Fig 1).

## Materials and Methods

### Location and field site

No specific permissions were required to conduct the field experiments nor to collect biological/water samples in Ria Formosa coastal lagoon, according to the national legislation, when the survey was conducted. Our study did not involve any endangered or protected species. Sampling was conducted in the Ria Formosa lagoon along the shore of Faro and Olhão, southern Portugal The Ria Formosa is a 55 km long shallow mesotidal lagoon with a surface area of approximately 16,300 ha. It forms the main nursery and source of recruitment of juvenile (economically) important fish species in the region [31]. It has five dune barriers, one artificial inlet, and two peninsulas that separate it from the open ocean [32]. The lagoon is relatively shallow and largely consists of salt marshes and shallow channels. Seawater circulation takes place through the six openings to the sea, the tidal range (between 1.5 and 3.7 m) and the shallow depths [33], by which between 50 and 75% of the water in the lagoon is exchanged daily by the tides [34]. The lagoon is considered a coastal water body by the European Water Framework Directive due to its salinity and water exchange with the ocean (Fig 2).

### Sampling of lagoon water and *Ulva* spp.

Subsurface seawater was sampled in the Ria Formosa lagoon on March 15^th^ 2010, on May 16^th^ 2011 and on March 12^th^ 2012 (Fig 2, Table 1). Over the three years, 22 surface water samples from different sites including three tidal pools and control samples were collected in triplicate (n_total_ = 66):

**Table 1:** Sampling sites and respective metadata.

Sampling spot Description	GPS coordinates in degrees decimal minutes	Water depth in m	Temperaturein°C	*p*H	Salinity in ‰	O_2_ in mg/L	Time	MG inducing activity like the factors of MS2 / MS6
**Sampling year: March 2010**								
**Ramalhete—Tidal pool 1**	N37°00.269’ W 7°57.851’	0.1	21.0	8.0	39.0	n.d.	11:30	**+/+**
**Ramalhete—Tidal pool 2**	N37°00.269’ W 7°57.851’	0.2	20.0	8.0	39.0	n.d.	11:40	**+/+**
**Ramalhete—Tidal pool 3**	N37°00.269’ W 7°57.851’	0.1	22.0	8.0	39.0	n.d	11:50	**+/+**
**Sampling year: May 2011**								
**Out of the lagoon**	N36°57.701’ W 7°57.214’	35	21	8.0	35.2	n.d.	n.d.	**-/-**
**1 “Marina praia do Faro”**	N37°00.156’ W 7°58.864’	2–3	22.1	7.98	36.7	4.63	14:02	**+/+**
**2 “Barinha”**	N36°58.978’ W 7°57.188’	1.5	20.9	7.98	36.9	4.60	14:20	**+/-**
**3 “Ramalhete—sea side”**	N37°00.269’ W 7°57.851’	4.7	23.1	8.02	37.6	5.02	14:30	**+/+**
**4 “In front of the train station Faro”**	N37°01.221’ W 7°57.031’	4.5	21.6	7.96	37.0	4.32	14:45	**+/-**
**5 “Wastewater treatment plant”**	N37°00.283’ W 7°54.536’	3.0	21.6	7.77	37.0	6.23	15:05	**+/-**
**6 “Entrance of water”**	N36°57.883’ W 7°52.202’	7.0	21.1	7.98	37.5	5.80	15:20	**+/-**
**7 “Between the two main channels”**	N36°58.679’ W 7°52.135’	6.2	20.8	7.98	37.5	5.55	15:25	**+/-**
**8 “Recovo Culatra”**	N36°59.402’ W 7°50.213’	1.2	23.2	8.04	37.2	5.40	15:40	**+/+**
**9 “Culatra sandbank”**	N37°00.187’ W 7°49.330’	2.4	22.1	8.10	37.1	4.64	15:50	**+/-**
**10 “Parque Natural”**	N37°01.699’ W 7°49.178’	5.0	22.1	8.02	37.1	4.60	16:05	**+/-**
**11 “Marina Olhão”**	N37°01.321’ W 7°50.851’	4.3	22.7	8.06	37.3	4.49	16:15	**+/+**
**Sampling year: March 2012**								
**Out of the lagoon**	N36°57.701’ W 7°57.214’	35	21	8.0	35.3	n.d.	14:10	**-/-**
**1**	N37°00.021’ W 7°56.210’	5.5	21	8.0	35.4	n.d.	14:35	**+/+**
**2 “Ramalhete—sea side”**	N37°00.395’ W 7°57.618’	2.5	22	8.0	35.7	n.d.	15:05	**+/+**
**3**	N37°00.051’ W 7°54.226’	3.0	21	8.0	35.4	n.d.	15:40	**+/+**
**4**	N37°00.021’ W 7°53.427’	1.0	21	8.0	34.6	n.d.	15:52	**+/+**
**5 “Ramalhete—tidal pool”**	N37°00.269’ W 7°57.851’	1.0	21	8.0	35.0	n.d.	11:00	**+/+**
**6 “Near the bridge”**	N37° 0.500’ W 7° 59.686’	0.3	21	8.0	35.0	n.d.	14:35	**+/+**

Water depth, temperature, pH, salinity, O_2_, and sampling time as well as morphogenetic activity (**MG**) equal to the activity of MS2-factor and MS6-factor were measured. In 2012, not all data were determined (n.d.) or were measured with less accuracy (i.e., pH, temperature). A brief definition of the sampling site is given in some cases.

In March 2010, seawater and *U*. *rigida* (GenBank KJ417445) were collected [35] from the three tidal pools #1–3 close to the Ramalhete Marine Station for analyses of morphogenetic activity and PCR-DGGE bacterial community profiling (see S1 Fig and S1 Appendix for details on PCR-DGGE methodology and results). In addition, bacteria were isolated from *U*. *rigida* surfaces collected from these tidal pools for the morphogenetic *Ulva* bioassay array (see below). The phylogeny of the *Ulva* species was determined according to Alsufyani et al. (2014).

In May 2011, subsurface water was taken from 11 sample sites to survey the morphogenetic activity across the lagoon along with a control sample outside the lagoon.

In April 2012, subsurface water was taken from 6 sample sites for a qualitative repetition of the survey conducted in 2011. One control sample was taken outside the lagoon again.

At each sample site, triplicates of 50 mL water samples were collected with sterile screw cap polypropylene tubes (BD Falcon, Biosciences, Germany), transported on ice to the laboratory and immediately sterile filtered twice (0.2 μm, polyethersulfone (PES), Roth, Germany) into a sterile tube cabinet under strictly sterile conditions in a laminar flow cabinet. The axenicity of the filtered samples was proven by PCR using the primers and cycling conditions described by Spoerner et al. (2012) [11]. The sterile-filtered seawater was frozen and stored at -80°C for the morphogenesis bioassays. The pH, oxygen saturation and temperature of the seawater were determined with a portable multi-parameter meter (Merck, Darmstadt, Germany) at each sampling site (Table 1).

### PCR-DGGE fingerprinting of bacterial communities on algal surfaces and in tidal pool water

To determine whether the surface of macroalgae found in the Ria Formosa tidal pools constitute a specific microhabitat for bacteria, we performed PCR-DGGE fingerprinting of algal epibacterial communities and their surrounding pool water. PCR-DGGE analysis also served to verify if bands observed in algal surface fingerprints matched the electrophoretic mobility of bacterial strains with morphogenetic activity, previously isolated by Spoerner et al. [11] from *U*. *mutabilis* in the laboratory. Details on PCR-DGGE methodology and main results are compiled in S1 Appendix.

### Isolation, molecular identification and phylogeny of *Ulva* surface-associated bacteria

For the isolation of surface-associated bacteria from *U*. *rigida*, 1 × 1 cm pieces of the algae collected from tidal pools #1–3 in 2010 (Table 1) were directly streaked onto Marine Agar (MA: DIFCO Marine Broth 2216, USA, supplemented with 1.5% agar). MA plates were incubated at room temperature (21°C) for 7 days, after which distinct colonies were picked and transferred with sterile toothpicks into 96 well cultivation plates containing 200 μL DIFCO Marine Broth 2216 in each well. Plates were then allowed to incubate for 5 days at room temperature (21°C), after which aliquots (100 μL) were taken for genomic DNA extraction using the DNeasy Blood & Tissue Kit (Qiagen, Hilden, Germany) following the manufacturer’s instructions. The remaining cell suspensions were stored in 15% glycerol at -80°C. Taxonomic classification of the bacterial isolates was performed by 16S rRNA gene sequencing. Nearly complete 16S rRNA gene fragments were PCR-amplified from genomic DNA using the primer pair 27f (ggg ttt gat cct ggc tca g) / 1390r (acg ggc ggt gtg trc aa) [36–38]. Reaction mixtures (50 μL) were prepared with 1 μL (50 ng) genomic DNA template, 37.5 μl H_2_O, and 5 μl 10× buffer (100 mmol L^−1^ Tris/HCl pH 8.3, 500 mmol L^−1^ KCl, 15 mmol L^−1^ MgCl_2_). Thermal cycling began with an initial denaturation step of 94°C for 5 min followed by 30 cycles of 94°C for 1 min (denaturation), 60°C for 1 min (annealing), 72°C for 2 min (extension) and a final extension step of 72°C for 10 min. Amplicons were then subjected to forward and reverse primer sequencing using the chain termination method (GATC, Göttingen Germany). Taxonomic classification and phylogenetic inference of bacterial isolates followed well established procedures with slight modifications [39,40]. First, sequences were manually trimmed using Sequence Scanner v1.0 software (Applied Biosystems, CA, USA) and assembled using DNABaser 3.5.0 (http://www.dnabaser.com/). Taxonomic assignment up to the genus level, when possible, was subsequently performed using the classifier tool of the Ribosomal Database Project (RDP, release 10, http://rdp.cme.msu.edu) at an 80% confidence threshold. Bacterial type strains representing the closest 16S rRNA gene relatives to the sequences generated in this study were identified using the RDP sequence match tool and included in phylogenetic inference for taxonomic reliability. For phylogenetic reconstructions, 16S rRNA gene sequences from our isolates, their closest type strains and reference *Ulva*-associated strains were aligned with the SINA web aligner tool within the ARB-SILVA database (http://www.arb-silva.de/aligner/). Alignments were imported into the ARB software and manually corrected using the ARB sequence editor window. The final FASTA format alignment was used as an input file for phylogenetic inference with the Maximum Likelihood (ML) method under the general-time reversible (GTR) evolutionary model with a discrete gamma-distribution of among-site rate variation (Γ4) and a proportion of invariant sites (I) using the software package MEGA version 6. The GTR-Γ4-I model was deemed the best-fit nucleotide substitution model for the dataset according to a preliminary model prediction run on MEGA 6. Sequences retrieved in this study were deposited in the European Molecular Biology Laboratory (EMBL) database under the accession numbers LN681258-LN681315. As an internal and quality control sample (QCS) of the sequencing, the morphogenetic active strains *Roseobacter* sp. strain MS2 (GenBank EU359909), *Cytophaga* sp. strain MS6 (GenBank EU359911), *Halomonas* sp. strain MS1 (GenBank EU359908), *Sulfitobacter* sp strain MS3 (GenBank EU359910), and the type strain *Cellulophaga lytica* (GenBank CP002534) were used.

### Cultivation of *U*. *mutabilis*

The mutant slender (sl-G(mt+)) strains of *U*. *mutabilis* [22,41] were cultivated and propagated in the absence (or presence) of bacteria in UCM (*Ulva* culture medium) under standard growth conditions [42,43]. Sterile culture flasks with gas-permeable screw caps (filling volume 20 mL; Nunc Int., Denmark) or standard Petri dish vessels were inoculated with freshly prepared axenic gametes (100 gametes / mL) for propagation of the gametophytes.

### Preparation of axenic cultures and the “*Ulva* bioassay array”

Gametogenesis of mature *Ulva* was induced by chopping and washing the algal fragments [42,43]. Three days later, gametangia were discharged by removal of a swarming inhibitor (SWI) through growth medium exchange [43]. Released gametes were purified from bacteria within a laminar flow cabinet with sterile equipment based on a slightly modified protocol by Spoerner et al. (2012) [11]: A concentrated solution of gametes was transferred into the wide end of a horizontally positioned Pasteur pipette (23 cm) filled with sterile UCM and the narrow end was directed towards a light source. Once the fastest gametes moved towards the narrow light-exposed tip, they were transferred into a new sterile Pasteur pipette. This procedure was repeated three times to obtain axenic gametes [17]. The stock solution of axenic gametes was diluted with UCM or the respective sterile-filtered lagoon water to reach the final concentration of 20–40 settled gametes/well (200 μL) conducted in 96-well micro array plates. Alternatively, axenic gametes in UCM were inoculated with the bacterial cultures isolated from *Ulva*. The density of gametes in the axenic stock solution was determined in 50% glycerol (v:v) using the Neubauer improved counting chamber [11]. Axenicity of the purified gametes was proven by PCR using the primers and cycling conditions described by Spoerner et al. (2012) [11]. After inoculation gametes settled within 24 h in the darkness and could be then used for the *Ulva* bioassay array.

### Survey of algal morphogenesis *via* the “*Ulva* bioassay array”

For testing the morphogenetic activities of the lagoon water, UCM was replaced with the sterile-filtered water samples. If necessary, salinity was adjusted by mixing with doubly concentrated UCM. Samples were applied to the bioassay array in a dilution series. The dilution was prepared with UCM to assure no shortage of nutrients over time. Dilution hereby refers to the total volume [x: (x+y), with x equal to parts of the collected seawater and y equal to the parts of UCM]. To avoid differences between several experimental setups and to assure comparable conditions, positive and negative controls were applied on the same multiwell plate. For positive controls, three wells were inoculated with strains MS2 or MS6 or both of them in each tested multiwell plate (Fig 1B).

For testing the morphogenetic activities of *Ulva*-associated bacteria, axenic gametes were inoculated with the isolated bacterial cultures (see above), which were first washed and re-suspended in sterile UCM. The calculated optical density in the well of the “*Ulva* bioassay array” was 10^−6^ upon dilution of the stock culture harvested at OD = 1.0.

After inoculation of gametes with sterile lagoon water or pure bacterial cultures, multiwell plates were covered with gas permeable sealing (Breathe-Easy, Diversified Biotech, MA, USA) to prevent contamination and placed into culture chambers at 20°C. The illumination regime was 17 hours light and 7 hours dark at 40–80 μmol photons m^−2^ s^−1^ as described by Wichard and Oertel (2010).

Growth and morphogenesis were carefully estimated under an inverted microscope (Leica, Wetzlar, Germany) by counting the cell numbers and observing the cell wall and rhizoid formation of 20 germlings per well. In particular, cell numbers were counted 14 days after inoculation and the percentage of algae with a normal cell wall was evaluated 7 days after the first cell wall deformation was observed in axenic control cultures. The experiment was conducted with biological triplicates. As the statistical deviation between (= germlings of different wells) and within (= germlings of the same well) the biological triplicates of the same treatment did not differ significantly (*p* > 0.5), the 95% confidence interval could be calculated based on the total sample size of n = 60 germlings.

Moreover, algal morphotypes were categorized after 3 weeks of cultivation according to the four known (described in Fig 1) and respective new groups.

## Results and Discussion

### Experimental design

*U*. *mutabilis* can be stably cultivated in laboratory conditions starting with a feedstock of axenic gametes purified without using antibiotics [43]. Detrimental side effects on *Ulva* can be thus ruled out [11]. However, to carry out representative and explorative field surveys of morphogenetic activities in natural seawater samples and bacterial growth media, we have further improved the cultivation regime by developing a simplified standardized bioassay in the scale of a 96-multiwell plate to enable large screenings [17]. A readily deployable *Ulva* bioassay array was developed based on previous experience with the tripartite *U*. *mutabilis*-*Roseobacter*-*Cytophaga* community (Fig 1A) [11,17], allowing us to survey morphogenetic activities under controlled conditions including positive and negative controls (Fig 1, see Material and Methods section). In particular, algal gametes can be obtained from the same cultural batch of *U*. *mutabilis* using germ cells of several individuals or even from a single individual *Ulva* specimen. Therefore, these standardized bioassays provide now robust conditions for monitoring the algal morphogenesis induced by waterborne compounds and/or bacteria. Variations in individual fitness or even slight differences in the growth condition (e.g., through changes in light, aeration, temperature and available nutrients), could have occurred if experiments and controls were set up separately, which was avoided. With our improved, miniaturized bioassays, we were able to screen more biological replicates and assess the dilution series to determine the arbitrary units of the potential morphogenetic activity in seawater samples. Considering the tedious and time-consuming work that such bioassays demand, the standardization of the 96 multiwell plates for individual *Ulva* cultures of germlings was essential to study the bacteria-induced morphogenesis.

For evaluation of the morphogenetic effects of the lagoon water samples and the isolated bacteria, we distinguished primarily between different growth activities previously described in the tripartite community of *U*. *mutabilis* [11] (Fig 1A):

- Morphotype I: In the absence of any morphogenetic compounds calloid development is caused and cell wall distortions such as bubble-like protrusions are formed.
- Morphotype II: Algal growth is promoted through cell divisions, but cell wall distortions are still visible (e.g., in the presence of strain MS2 only).
- Morphotype III: Normal cell wall development is combined with the formation of a rhizoid (e.g., in the presence of strain MS6 only), but no blade is formed.
- Morphotype IV: Synergistic effects of both factors result in normal growth and morphogenesis towards an adult *Ulva*.

### Morphogenetic activity of water samples from tidal pools

Tidal pools are potential sources of chemically enriched water because released compounds by organisms living in this habitat can accumulate in between the (spring) tide and thus abundance studies might easily provide information on the potential morphogenetic activities in natural samples. The investigated sandy tidal pools #1–3 were 10–15 cm deep when samples were taken (Fig 2, blue dot). Water samples were collected from tidal pools just before they were flooded by high tides. For comparison, water samples were also taken from one of the main channels of the lagoon, which did not become dry during the low tide.

**Fig 3:**
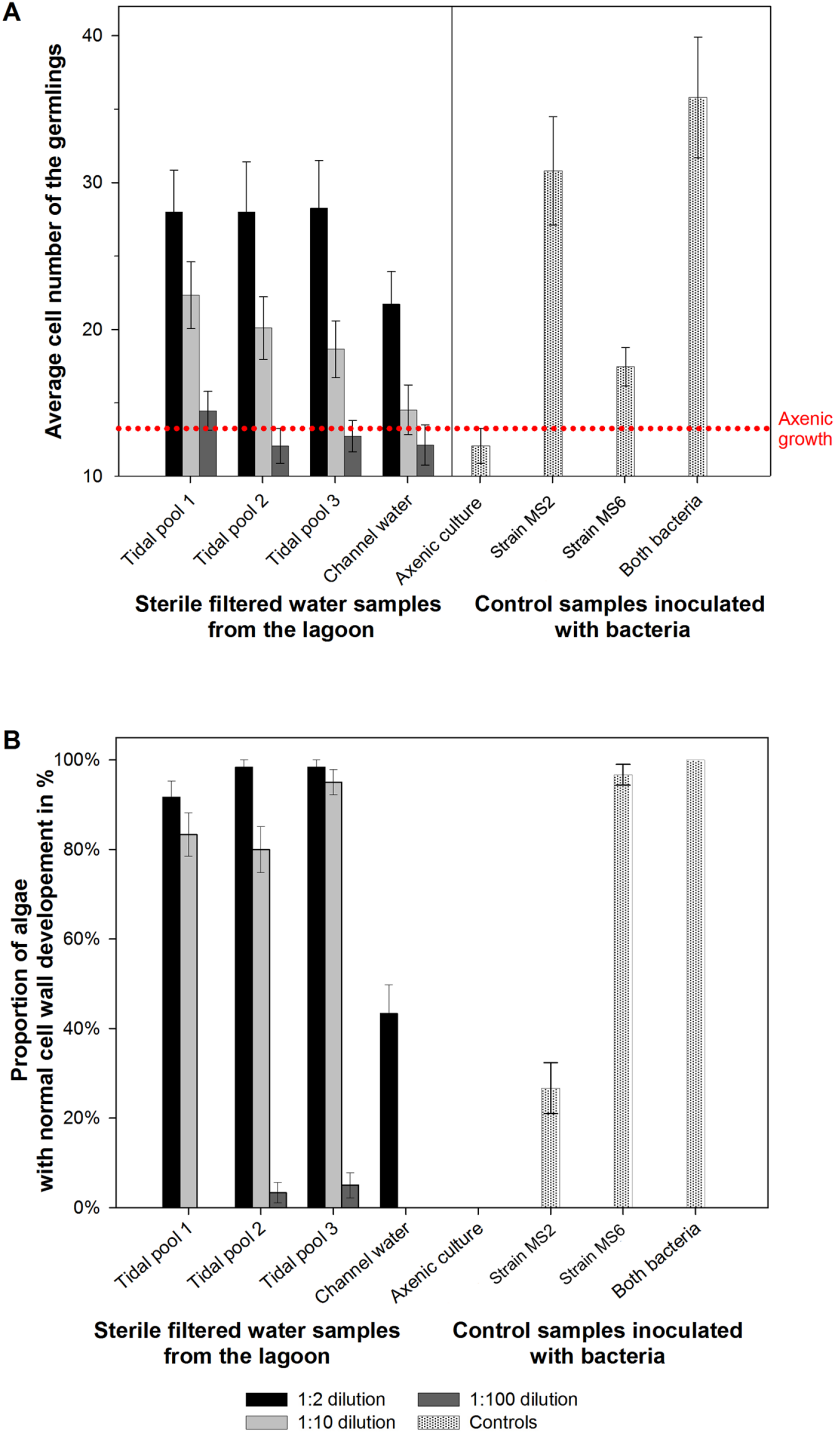
Bioassay of the morphogenetic activity with doubly sterile-filtered water from tidal pools and a main channel of the lagoon. **(A)**: Dilution series of the seawater samples with UCM were tested to estimate the potential morphogenetic activity on axenic *Ulva* gametes. Controls show the morphogenetic activity on gametes without bacteria, with the bacterial strain MS2, with the bacterial strain MS6 and the completely resembled morphology with both strains (MS2 and MS6). To estimate the MS2-like activity, the cell numbers of the germlings were counted 14 days after inoculation. (**B**) To estimate the MS6*-*like activity, the percentage of algae with a normal cell wall was evaluated 7 days after the first cell wall deformation was observed in axenic control cultures. Error bars represent (**A**) confidential intervals (P = 0.95; n = 60 individual algae) or (**B**) standard deviations (n = 60 individual algae). The dotted line indicates the maximum growth and development under axenic conditions.

Morphogenetic effects of sterile-filtered seawater samples were found in all investigated tidal pools (Figs 2 and 3). The activity expressed by the number of cells and the proper cell wall formation of germlings did not significantly differ between the water samples of the tidal pools, despite the fact that the pools were being populated with different algal communities: *Ulva rigida* in tidal pool #1, *Ulva rigida*, *Fucus vesiculosus* and *Blidingia* sp. in tidal pool #2 and *Blidingia* sp. in tidal pool #3. In contrast, the samples collected from the channel of the lagoon showed only a moderate morphogenetic activity (Fig 3A).

Using a dilution series of the seawater with UCM, the morphogenetic activities of the collected samples were estimated. Morphogenetic compounds could be found in excess in each investigated tidal pool, as seawater samples diluted up to 9 parts of UCM (i.e., 1:10) still revealed a significant induction of cell division recorded by the numbers of *Ulva* cells counted per well (Fig 3A). The mode of action resembled exactly the cytokinin-like activity of the known MS2-factor [11], as indicated by the control experiments conducted in parallel. The morphogenetic activities of tidal pool water decreased with increasing dilution and vanished completely with a dilution of 1:100, at which the concentration gametes developed consistently into a callus-like morphotype.

A pronounced effect was also observed for the auxin-like growth factor originally found in strain MS6 [11] (Fig 3B). Identical samples from all tidal pools had a positive effect on the cell wall and rhizoid formation of *U*. *mutabilis* at a dilution of 1:2 and even at 1:10 meaning that a complete algal morphogenesis was observed upon addition of sterile-filtered seawater to axenic gametes. However, no effect was observed at a dilution of 1:100. Samples from the water body of the channel revealed a weak effect on cell wall formation, even in diluted samples. Overall the observations suggest that free-living bacteria or surface-associated bacteria even from other macroalgae rather than *Ulva* species may also have morphogenetic activity. Waterborne morphogenetic factors were even verifiable outside the tidal pools in the water body of the main channel. Differences between water-borne morphogenetic activities of tidal pool samples (1–3, blue spots) and seawater of the lagoon´s channels could be due to an accumulative effect in-between the tides.

### Determination of bacterial communities based on cultivation independent approaches

The most abundant *Ulva* species during the survey in the Ria Formosa, *U*. *rigida*, [35] was selected to determine its specific epibacterial community. The bacteria isolated from the algal surface were compared with those obtained from seawater samples in the vicinity of the algae. 16S rRNA gene-based PCR-DGGE fingerprinting demonstrated that epibacterial communities from algal surfaces were sharply distinct from their surrounding bacterioplankton (S1 Fig), providing further evidence for selective shaping of epibiotic consortia on macroalgae as observed elsewhere [44]. Intriguingly, none of the dominant PCR-DGGE bands observed in profiles of bacterial communities from algal surfaces matched the electrophoretic mobility of morphogenetically active bacteria isolated by Spoerner et al. (2012) from *U*. *mutabilis* in the laboratory (S1 Fig), prompting us to isolate bacteria from *Ulva* specimens *in situ*, and determine their morphogenetic capacities and phylogenetic affiliation. (For further details on PCR-DGGE methods and results, see S1 Appendix.)

### Molecular identification of *Ulva* associated bacteria and their morphogenetic activity

**Fig 4:**
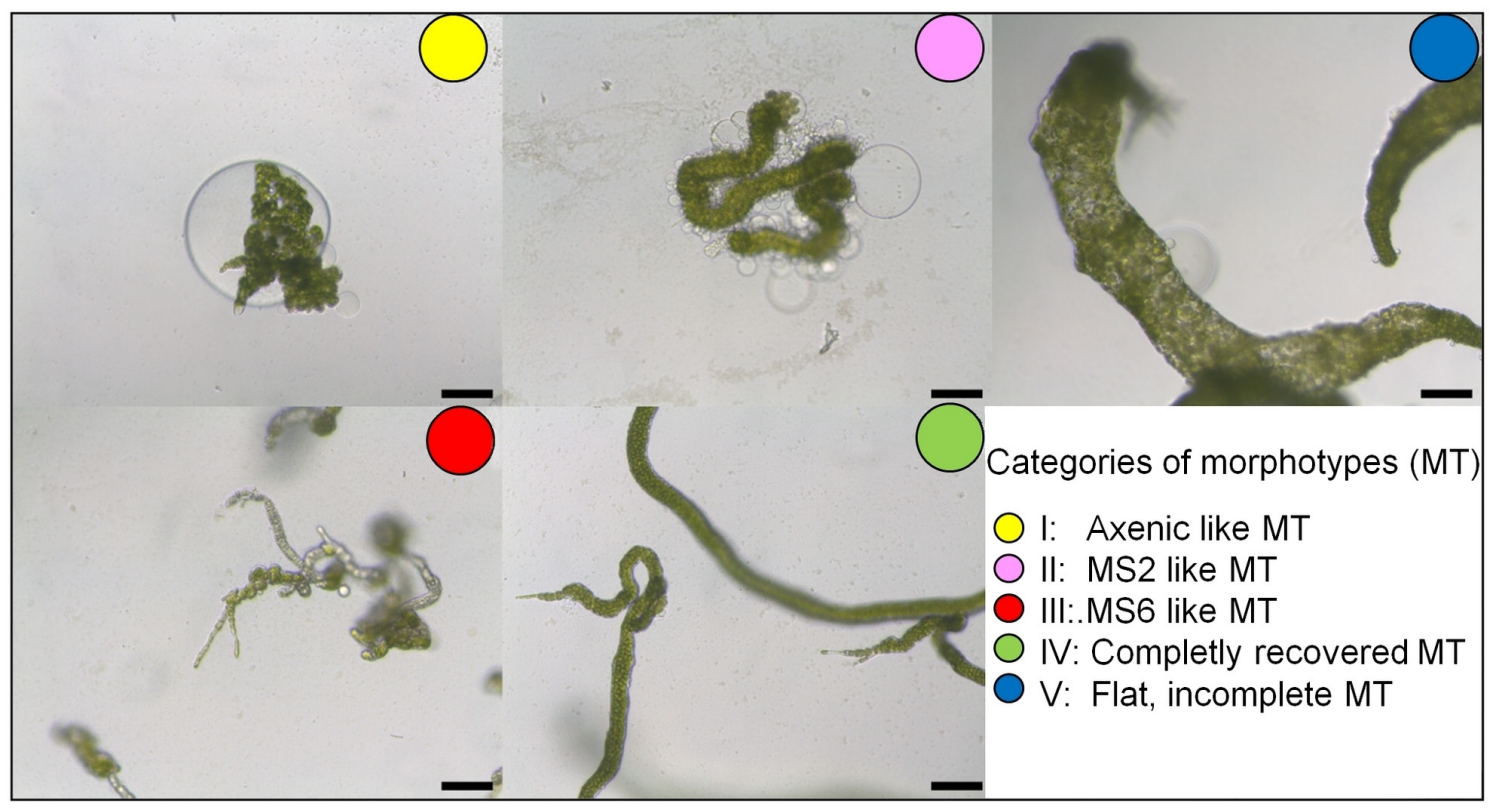
Screening of morphogenesis inducing bacteria in 96 microarray plates isolated from the surface of *U*. *rigida*. Various morphotypes were identified: Known morphotypes **I-IV** (as also found in the standard assays with strains MS2 and MS6) and the novel morphotype **V** with enlarged cells or vacuoles.

**Fig 5:**
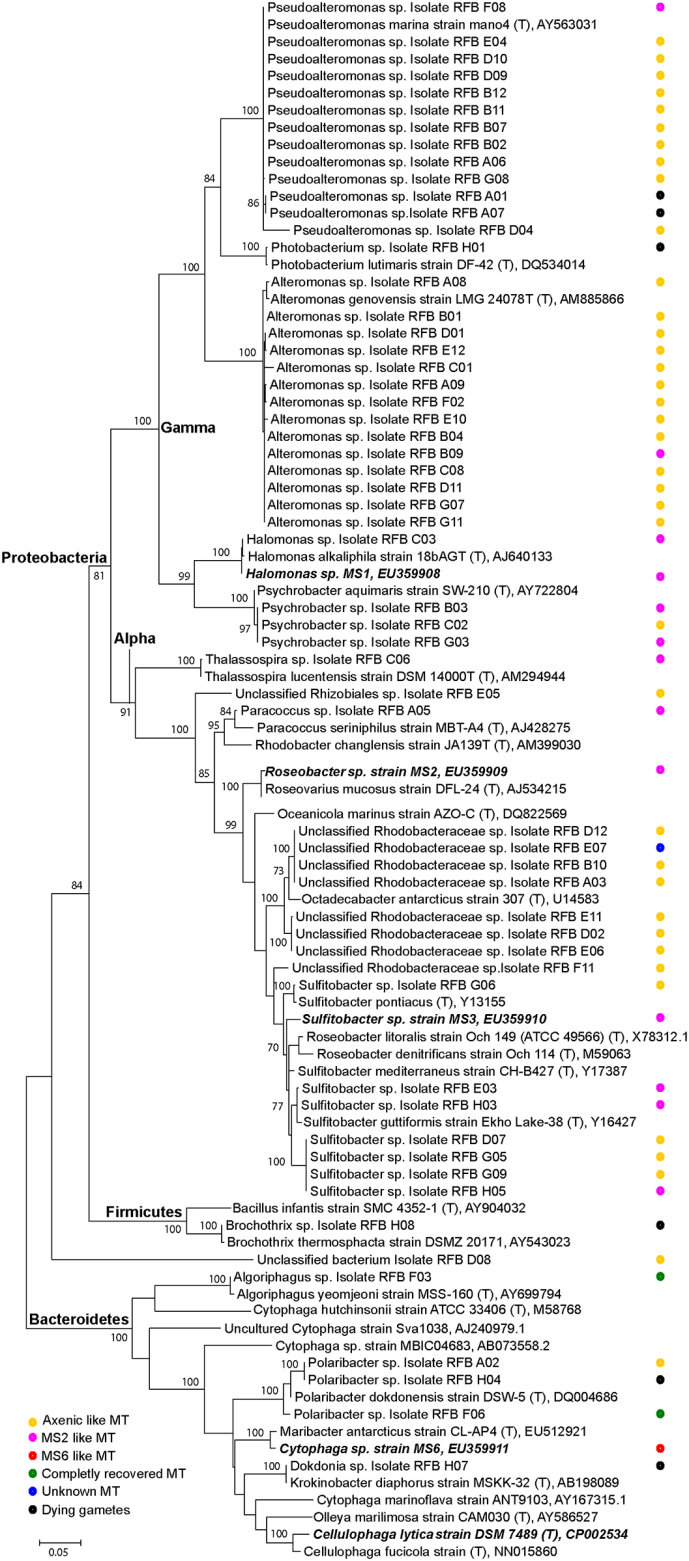
Phylogenetic tree of the isolated bacteria. Maximum likelihood phylogram of isolated and cultivatable bacteria inferred from the 16S rRNA gene. Bacteria were collected through swapping from the surface of *U*. *rigida* collected in tidal pools in 2011. *Halomonas* sp. MS1, *Roseobacter* sp. MS2, *Sulfitobacter* sp. MS3 and *Cytophaga* sp. MS6 were used as reference strains isolated from *U*. *mutabilis* [11]. The algal morphotypes induced by the bacteria are annotated.

High quality 16S rRNA gene sequences with no background peaks in the electrochromatogram, indicative of pure cultures, could be obtained for 57 colonies which were subsequently tested in the *Ulva* bioassay array for their morphogenetic activity. Thirty-eight of the 57 strains had no effect (morphotype I) and 10 were able to promote growth, but they did not elicit normal cell wall development (morphotype II). The same was true for one specific isolate that induced a particularly newly observed flattened morphotype (Figs 4 and 5, morphotype V). Most intriguingly, two isolates could solely induce complete algal morphogenesis (morphotype IV), which has not been observed previously, while six isolates had detrimental effects on algal development (Fig 5).

Out of the 57 isolates, 50 belonged to the phylum *Proteobacteria* (18 in the *Alpha-* and 32 in the *Gammaproteobacteria* classes), five belonged to the phylum *Bacteroidetes* and one to the phylum *Firmicutes*, whereas one isolate could not be assigned a bacterium phylum with the RDP classifier tool (Fig 5). In total, 47 isolates could be classified at the genus level based on 16S rRNA gene sequencing. The most represented genera were *Alteromonas*, *Pseudoalteromonas* and *Sulfitobacter*, each containing 14, 13 and 7 identified isolates, respectively, followed by *Psychrobacter* and *Polaribacter*, each containing 3 isolates. The genera *Photobacterium*, *Halomonas*, *Thalassospira*, *Paracoccus*, *Brochotrix*, *Algoriphagus* and *Dokdonia* were each represented by one single isolate. From the remaining 10 isolates, which were non-classifiable at the genus level, eight were affiliated with the family *Rhodobacteraceae* (*Alphaproteobacteria*) (seven of these were closely related to the genus *Octadecabacter*). Finally, one isolate was classified in the *Rhizobiales* order (*Alphaproteobacteria*) and one remained unclassified at the phylum level as mentioned above. Overall, RDP classifier results were highly congruent with phylogenetic inference, i.e., with the relatedness of our isolates with type strains of formally recognized genera, thus giving solid support to the attempted genus-level classifications. Furthermore, our phylogenetic assessment suggested that the morphogenesis-inducing strains MS2 and MS6, which were previously tentatively classified as *Roseobacter* sp. and *Cytophaga* sp. [11], in fact affiliated better with the genera *Roseovarius* and *Maribacter*, respectively, following the current bacterial nomenclature (Fig 5) [45–47].

Overall, the dominance of the phylum *Proteobacteria* within the cultivable bacteria obtained from *Ulva* surfaces, and macroalgae in general, supports previous findings with *Ulva* species [48]. The proteobacterial isolates, mainly *Rhodobacteraceae* and *Halomonadaceae*, induced the MS2- morphotype (Figs 4 and 5). The release of the MS2-factor is thus not genus dependent [11,17], although the strain MS2 can be chemotactically attracted by *U*. *mutabilis* [11], indicating an active recruitment process. Interestingly, a high frequency of neutral results (i.d. “axenic-like” gametes: morphotype I) was also observed for members of this phylum. The *Pseudoalteromanonas* and *Alteromonas* strains did not show any morphogenetic activity with few exceptions, indicating isolate-specific activity e.g., RFB F08. Besides the apparently asymbiotic and symbiotic effects, there are evidences that bacteria, for example certain *Pseudoalteromonas* sp. (RFB A01, A07) and *Bacteroidetes* isolates, had a potent algicidal effect on gametes and germlings of *Ulva* (Fig 5). Interestingly, it was earlier reported that *Pseudoalteromonas* isolates can potentially regulate the termination of harmful algal blooms such as of *Gymnodinium* via released toxic compounds [49]. The two strains inducing complete morphogenesis belonged to the *Bacteroidetes* phylum, namely RFB F03 (*Algoriphagus* sp.) and RFB F06 (*Polaribacter* sp.) (Fig 5). Genome analysis of e.g. *Polaribacter* sp. MED152 revealed genes for e.g. attachment to surfaces and polymer degradation [50]. This agrees with the assumed life strategy of marine Bacteroidetes [47] and the previously identified MS6 strain associated to *U*. *mutabilis* [11]. In any case, the ratio between the different morphotype-stimulating and inactive bacteria cannot be exactly determined because only cultivatable bacteria could be used in the bioassay, and the majority of bacteria naturally occurring on *Ulva* [e.g., 44] could certainly not be successfully isolated [51].

### Prevalence of the waterborne morphogenetic compounds in the Ria Formosa lagoon

**Fig 6:**
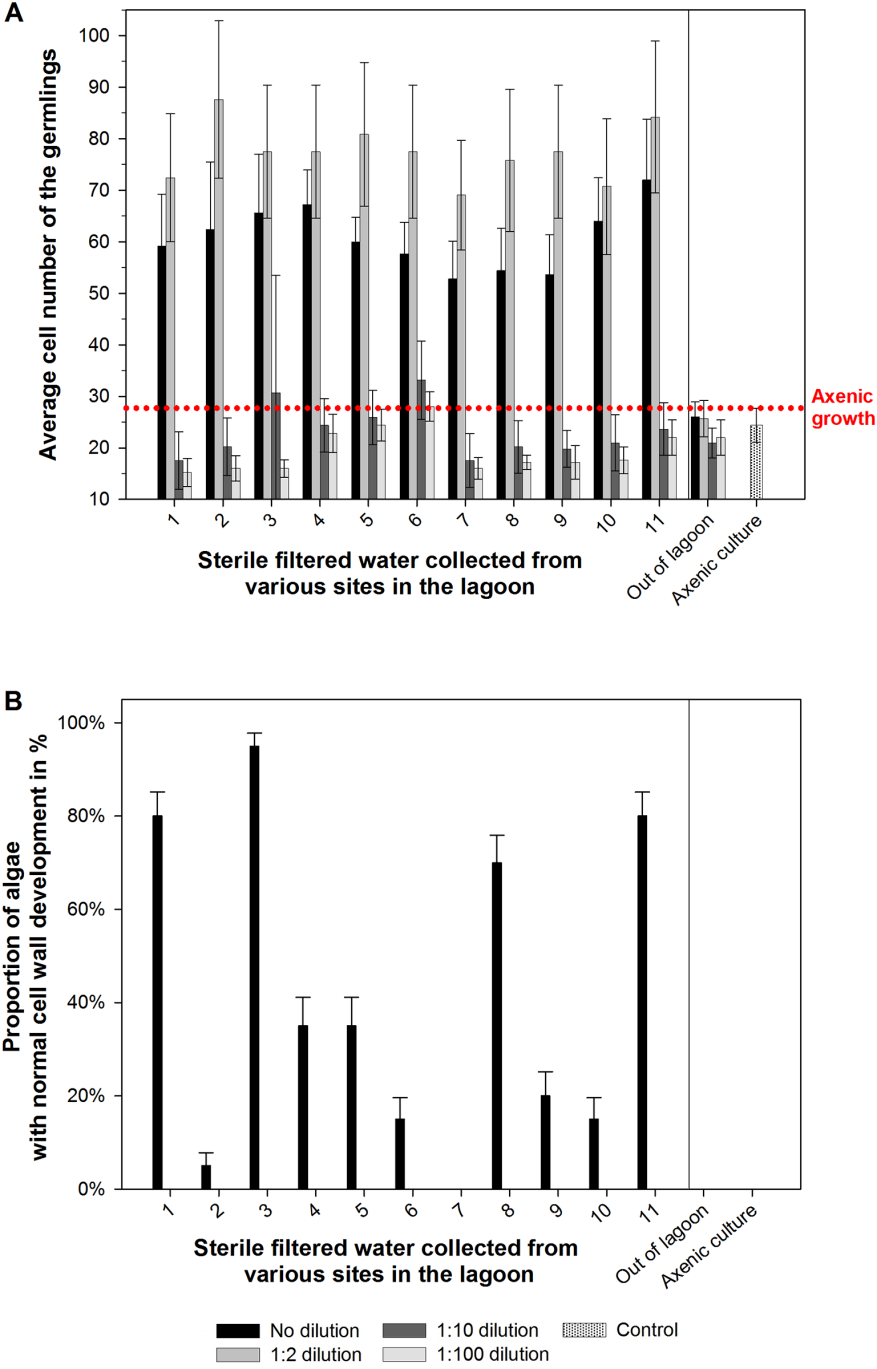
Bioassay of the morphogenetic activity with doubly sterile-filtered water collected from various sampling sites across the lagoon. (**A**) A dilution series of the seawater collected in 2011 with UCM was tested to estimate the potential morphogenetic activity on axenic *Ulva* gametes. The control of axenic growth is shown for comparison. To estimate the MS2-like activity, the cell numbers of the germlings were counted 14 days after inoculation. (**B**) To estimate the MS6-like activity, the percentage of algae with a normal cell wall was evaluated 7 days after the first cell wall deformation was observed in axenic control cultures. Error bars represent (**A**) confidential intervals (P = 0.95; n = 60 individual algae) or (**B**) standard deviations (n = 60 individual algae). The dotted line indicates the maximum growth and development under axenic conditions.

To survey the prevalence of morphogenetic compounds released into the seawater of the Ria Formosa lagoon, explorative studies became very valuable. Subsurface seawater samples were taken from 16 diverse spots scattered throughout the lagoon in spring 2011 and 2012 covering a variety of locations accessible within a short time frame to avoid the dilutive influence of incoming high tides (Fig 2). The growth promoting factor (i.e., MS2-factor) was found at all sampling sites within the lagoon (dilution 1:2), but not outside of the lagoon (Figs 2 and 6). Upon further dilution by 1:10, the factor was not measureable at any tested sampling site as indicated by the axenic morphotype (Fig 6A). The homogeneous distribution of the MS2-factor, elicited by *Roseovarius* and closely related strains such as *Roseobacter* and *Sulfitobacter* highlights the ubiquity of members of the *Roseobacter/Rhodobacteraceae* clade which is equipped with a tremendous diversity of metabolic capabilities [52]. This in part explains their success in so many different marine habitats. In contrast to the homogeneous distribution of the MS2-factor, the prevalence of the MS6-factor (*Maribacter* sp., *Bacteroidetes*, see below) could be found only at a few sampling sites (Fig 6B). Four out of eleven water samples taken from different spots in 2011 were biologically active in the “*Ulva* bioassay array” and contributed to the normal cell wall development of *U*. *mutabilis* gametophytes. The sterile seawater samples from the other seven sampling sites within the lagoon had minor or absent (< 50% recovering of cell wall formation) effects on the elimination of cell wall distortions (Fig 6B, see e.g., sampling sites #2, 4–7, 9 and 10 in 2011; red dots), which are always observed in axenic cultures. Moreover, the effect of the MS6-factor completely vanished at any dilution and is hence not produced in elevated amounts. This impedes structural elucidation of these interesting natural products [11], which is still under investigation for *Ulva* [17], but have been already successfully established for *Monostroma oxyspermum*, when the structure of the morphogen thallusin was revealed [10].

Notably, undiluted samples provided sometimes slightly less growth promoting activity than the samples diluted with the UCM (ratio 1:2, Fig 6A). As the UCM has been always used for all dilution series as well as in the control cultures, morphogenetic effects of the medium can be excluded, as also demonstrated by Spoerner et al. (2012) and Matsuo et al. (2005). The slower growth in undiluted seawater samples might be thus caused by a shortage of available essential nutrients. This shortage can be compensated by a vitamins enriched UCM (data not shown). In summary, sterile-filtered seawater contained all the ingredients including phytohormone-like morphogenetic compounds necessary to facilitate the complete morphogenesis of *U*. *mutabilis* during the timeframe of sampling. Similar to the classification used for the description of the algal morphogenesis-inducing activity of bacteria in *U*. *mutabilis* [11], axenic undiluted lagoon water samples can be categorized according their morphogenetic activity into the following four groups: seawater harboring (i) no morphogenetic activity, (ii) morphogenetic activity similar to the MS2-factor, (iii) morphogenetic activity similar to the MS6-factor, and seawater samples (iv) inducing the complete morphogenesis of *U*. *mutabilis*.

Noteworthy the MS2-factor could be found at all sampling sites, whereas the MS6-factor was not ubiquitously distributed over all sampling sites in 2011. In 2012, the survey was repeated qualitatively with fewer sampling spots and resulted in comparable findings. (Table 1, Fig 2, sample sites #1–6; green dots).

The homogenous distribution of the MS2-factor corresponds most likely to multiple bacterial sources scattered in the lagoon. Indeed, several previous studies have already suggested that growth promoting and morphogenesis inducing compounds can be released by various bacterial species independent of their phyla including *Proteobacteria*, *Firmicutes* and *Bacteroidetes* [8,9,11,14,16–18,53]. Hereby, the molecular nature of these activities was demonstrated in the chemosphere of the tripartite community between *U*. *mutabilis* and its two associated bacteria [11].

The patchy distribution of the MS6-factors in the lagoon implicated a specific source, in particular, when the factor was at least 10 times more concentrated than the necessary biological threshold concentration. The MS6-factor was up to now only found to be produced by bacteria belonging to the *Bacteroidetes* phylum [11], which are often living epiphytically on algal surfaces [54,55]. It is thus tempting to speculate that spots of elevated amounts of these morphogens are presumably dominated by *Ulva* (e.g., sampling site #3 in 2011) as observed for the tidal pools in 2010, but this needs to be the objective of further studies.

Overall, the most intriguing finding that seawater samples can completely recover the morphogenesis of axenic germlings again supports previous observations that a direct cell-cell-contact [15] is not necessary for the bacteria-induced morphogenesis of *Ulva* [10,11]. Interestingly, both MS2 and MS6-factors accumulated over six weeks in land-based *Ulva* aquacultures starting with morphogen-free artificial seawater (Instant Ocean^®^) using large scale closed cultivation systems without growth medium exchange (Grueneberg and Wichard, unpublished data). A rapid decomposition of both morphogenetic factors can be thus ruled out because all active water samples were biologically vigorous for the prolonged time as tested by the “*Ulva* bioassay array”. To verify this observation in the field, future time lapse measurements of the morphogenetic compounds are necessary. Moreover, wastewater effluents from land based *Ulva* aquacultures containing the MS6*-*factor might support the development of *Ulva* propagules and trigger sequentially the green tide formation in coastal areas.

Contrariwise marine stations provide usually (sterile-) filtered seawater for algal cultivation used for biomass production but also for sensitive experiments in marine ecology. Here, waterborne morphogenetic compounds might interfere with the goal of having stable and reproducible land-based *Ulva* cultures as the amount of morphogenesis-promoting factors might vary strongly and thus alter the growth conditions by using lagoon water. Easily available seawater from the lagoon, enriched with morphogenetic compounds, could certainly be also used to promote growth of an axenic feedstock of *U*. *mutabilis* in land-based aquacultures.

### Ecophysiological functioning of *Ulva*´s morphogenesis in its bacterial environment

The strong prevalence of the MS2-factor in the Ria Formosa might be caused by several unspecific bacterial sources and the good miscibility of the factor within the lagoon. The assemblage of epibacteria-mediated morphogenesis revealed that the activity of strain MS2 was ubiquitous in two different ways: In the first place, the ecophysiological functions can be overcome by a variety of bacteria from at least two classes, Alpha- and Gamma-proteobacteria, and second, the activity was not directed to a specific *Ulva* species. In fact, epibacteria isolated from *U*. *rigida* can promote cell division in *U*. *mutabilis*, as bacteria isolated from *U*. *mutabilis* do for *U*. *linza* [53] and vice versa (data not shown). Due to the ubiquity of the diffusible morphogenetic factor in the lagoon, the presence of specific bacteria in *Ulva*´s vicinity does not directly matter as long as they are able to release the respective morphogenetic compounds, which can then be perceived by *Ulva*. From this point of view, the availability of the waterborne and ecophysiological functioning morphogens matter more than the immediate presence of epiphytic bacteria on *Ulva*. Indeed, this implies that *Ulva* does not need to select for specific bacteria producing the MS2-factor, although controlled mutualistic interactions between the MS2 strain and *U*. *mutabilis* have already been described [11]. Our results thus partly support the hypothesis of a competitive lottery by Burke et al. (2011) [44,56], who argue that ecological niches e.g., *Ulva*´s surface, are randomly colonized with coexisting bacteria of similar ecological (i.e., ecophysiological) functions. This finding also implies that the composition of bacterial species is determined randomly by recruitment from bacterial guilds, whose members are functionally equivalent (i.e., releasing the MS2-factors). However, considering that algal surface chemistry can control bacterial biofilm formation [28], selective and mutualistic processes have to be followed as well. In this context, the MS6*-*factors, which promotes rhizoid growth and allows normal cell wall development, was rare among the bacterial isolates. Either this factor is limited to a much smaller number of bacterial strains, or the bacteria are restricted to those that are not cultivatable under our cultivation conditions. Bacterial growth medium supplemented with *Ulva* extracts might be promising for the successful cultivation of hard-to-domesticate bacteria in future studies. Bacteria provided with cell-wall degrading enzymes, such as the *Cytophaga*, may also permeate cell walls and thus become difficult to isolate [11]. Certainly, strain MS6 might be specifically associated with *U*. *mutabilis* and not with *U*. *rigida*. Moreover, it was shown that the growth of strain MS6 can be improved in the presence of both strain MS2 and *U*. *mutabilis*, clearly revealing the essential mutualistic interactions [11]. Although the morphogenesis-inducing activity of strain MS6 could not be replaced by any other bacteria isolated from *U*. *rigida* surfaces, ecological redundancy was surprisingly accomplished by two isolates (*Algoriphagus* sp. and *Polaribacter* sp.) which each solely induced the complete morphogenesis of *U*. *mutabilis*. These isolates are of particular interest as multiple ecophysiological functions are combined, and the tripartite community can be further reduced into a bipartite mutualistic interaction. A combined approach in chemical ecology and molecular biology will further explore the chemical structure of bacterial morphogens and their perception by *Ulva* [20,57].

## Conclusions

The ecological relevance of diffusible morphogenetic compounds originally observed in laboratory experiments for Ulvales were verified in natural subsurface seawater samples of the Ria Formosa lagoon. Environmental morphogenetic compounds play thus an essential role in the development of *Ulva* and potentially in the initiation and progression of green tides in a microbiome-dependent fashion. Whereas bacteria were clearly identified as a source of morphogenetic compounds, other organisms may also contribute to the pool of morphogenetic compounds perceived by *Ulva* in its biocoenose. Future studies need to clarify whether cross-talk also occurs between *Ulva* and other algae or sea grasses to affect growth and morphogenesis. The relative concentration of morphogenetic compounds was estimated by a dilution series indicating the very low concentrations but high biological activity of both the MS2- and MS6-factors. Despite the fact that we did not find any solely MS6-like effect within the group of the cultivable bacteria, *Ulva*´s morphogenesis was completely recovered with sterile-filtered lagoon´s water, which contained the complete chemical cocktail that *U*. *mutabilis* needs to ensure morphogenesis. A combined approach of metagenomic investigations of (epi)bacterial communities and morphogenetic bioassays of *Ulva* during various seasons and green tides should further improve our understanding of the ecological relevance of morphogenetic compounds in coastal seawater. Beyond the eco-physiological implications, the various effects of filtered seawater on algal development in land-based tanks have to be considered. Frequently used filtered seawater in scientific experiments and in commercial aquaculture can support the growth and development of *Ulva* depending on the morphogenetic inductive potential, and might directly influence the success of biomass production. This study revealed once more how signal-mediated cooperative interactions between kingdoms contribute to a functioning mutualism in *Ulva*´s chemosphere.

## Supporting Information

S1 Appendix Grueneberg et al. (PDF)

S1 Fig Bacterial fingerprinting based on the amplification of 16S rRNA. **(A)** DGGE shows the ribotypes of representative samples to survey the bacterial communities in seawater (W2-W8) and on algal surfaces (A10-A15). Lanes (S1, S9, and S15) represent a standard consisting of defined DNA of laboratory strains. Seawater samples have been taken from the following tidal pools (W2: pool #1; W3, 4, 5: pool #2; W6, 8: pool #3) and from a main channel of the lagoon (W7) in 2010. Bacterial analyzed were swabbed from the surface of *Ulva rigida* (A10: pool #1, A11: pool #2,), *Fucus vesiculosus* (A12: pool #2), *Blidingia* sp. (A13: pool #2, A14: pool #3) and from the seagrass *Zostera noltii* (A15) collected in a further tidal pool nearby. (**B**) The two origins of bacteria, seawater (turquoise circle) and algal/seagrass surface (black circle), were compared with each other based on the bacterial fingerprinting using unsupervised principal coordinate analysis (PCO) in order to visualize class distinctions. Axis 1 extracted 57% of the variation, axis 2 extracted 15% and axis 3 extracted 10%.
(TIF)
