# Supplementary material for: Macroalgal Morphogenesis Induced by Waterborne Compounds and Bacteria in Coastal Seawater
Source: PLoS One. 2016 Jan 8;11(1):e0146307. doi: 10.1371/journal.pone.0146307 (PMC4720170; doi:10.1371/journal.pone.0146307)
Supplement: S1 Appendix — (PDF) [file pone.0146307.s001.pdf]

Supporting Information for

Macroalgal morphogenesis induced by waterborne compounds and bacteria in coastal seawater

Jan Grueneberg[1], Aschwin H. Engelen[2], Rodrigo Costa[2], Thomas Wichard[1]*

[1] Institute for Inorganic and Analytical Chemistry, Jena School for Microbial Communication, Friedrich Schiller University Jena, Jena, Germany;

[2] Center of Marine Sciences, University of Algarve, Faro, Portugal

* Corresponding author:

E-mail: Thomas.Wichard@uni-jena.de,

Content:

1. PCR-DGGE methodology

1.1 Metagenomic DNA extraction

1.2 PCR-DGGE bacterial community fingerprinting

2. DGGE results

2.1 PCR-DGGE analysis for the identification of microbial populations on algal seaweeds

3. References

1. PCR-DGGE methodology

1.1 Metagenomic DNA extraction

Lagoon water and algal surface samples were collected for PCR-DGGE analysis to determine (i) whether epiphytic bacterial communities from tidal pools substantially differ from those of the surrounding pool water and (ii) whether PCR-DGGE bands from the algal fingerprints matched the electrophoretic mobility of bacterial strains previously isolated from *Ulva mutabilis* (see main text).

In total, six algal samples were taken from various tidal pools for this purpose, namely *Ulva rigida* (n=2), *Fucus vesiculosus* (n=1), *Blidingia* sp. (n=2) and *Zostera noltii* (n=1). Algae were taken to a laminar flow cabinet and one piece in the range of 1 × 1 to 2 × 2 cm per specimen (about 10 mg fresh weight) were transferred with sterile tweezers into 10 mL of sterile *Ulva* culture medium (UCM [1]), in which they were incubated for 5 min while shaking gently every minute. The washing step was repeated once with fresh sterile UCM. The washed pieces of the thallus were then swabbed with sterile swabs (Omni Swab, Whatman Bioscience, USA). The swab heads were transferred into a sterile 2 mL polypropylene microcentrifuge tube (Eppendorf, Wesseling-Berzdorf, Germany) and frozen at -80°C until DNA extraction. Seawater samples (50 mL) were passed through a sterile paper filter to remove bigger particles, organisms and debris. The sample was then passed through a sterile filter (0.2 µm, Isopore™ filter, type GTTP, Millipore, Schwalbach, Germany) using a polysulfone syringe filter holder assembly (Nalgene Nunc, USA). The filter was subsequently transferred with sterile tweezers into a sterile 2 mL polypropylene microcentrifuge tube and stored at -80 °C until DNA extraction. Metagenomic DNA was extracted from bacteria collected on filters (50 mL water samples) or on swabs (*Ulva*

surface) with the QIAamp DNA Mini Kit (Qiagen, Hilden, Germany) following the company's protocol for DNA purification from buccal swabs modified with an additional lysis step for gram-positive bacteria. The extracted DNA was finally eluted in 50 µL Tris-Cl / EDTA (10 mmol L^{-1} / 0.5 mmol L^{-1}, pH = 9) and stored at -80 °C.

1.2 PCR-DGGE bacterial community fingerprinting

Cultivation-independent analysis of bacterial community structure in *Ulva* surface and lagoon water samples was performed by PCR-DGGE fingerprinting of 16S rRNA gene fragments amplified from metagenomic DNA. To this end, the primer pair 358fGC (cgc ccg ccg cgc gcg gcg ggc ggg gcg ggg gca cgg ggg gcc tac ggg agg cag cag) and 907rM (ccg tca att cmt ttg agt tt) [2,3] was used to amplify a fragment of 550 bp spanning the V3 – V5 hyper variable regions of the 16S rRNA gene. The reaction mixture (50 µL) consisted of 1.5 µl bovine serum albumin (BSA, 20 mg L^{-1}), 1 µL dNTP mix (10 mmol L^{-1}), 2.5 U DreamTaq™ DNA Polymerase, 5 µL DreamTaq™ Buffer (10×), and 2 µL of each primer (10 mmol L^{-1}) and 20 ng template DNA. Reagents were purchased from Fermentas (St. Leon-Rot, Germany) and bovine serum albumin (BSA) was acquired from Sigma-Aldrich (Munich, Germany). PCR cycling conditions comprised an initial denaturation step at 94 °C for 5 min followed by 10 touchdown cycles, lowering the annealing temperature by 1°C each cycle beginning at 65 °C (1 min) and then 25 cycles with the annealing temperature of 55 °C, (1 min) and elongation at 72 °C (1 min) followed by a final extension step of 10 min at 72 °C. The resulting amplicons (200 ng) were loaded onto an 8% polyacrylamide gel with a denaturing gradient of 20 to 70% denaturants (100% denaturants contained 7 mol L^{-1} urea and 40% formamide) in a DCode electrophoresis system for DGGE (Bio-Rad

Laboratories, Munich, Germany). Electrophoresis was carried out in Tris-acetate-EDTA buffer (pH 7.8) for 12 h at 100 V and 60 °C. PCR products of the reference strains *Roseobacter* sp. MS2 (Genbank EU359909), *Cytophaga* sp. MS6 (Genbank EU359911), *Cytophaga* sp., *Micrococcus* sp. and *Pseudoalteromonas tetraodonis* were mixed and loaded at the edges and in the middle of the gel as markers [4]. The gel was stained with the fluorescent dye SYBR® Gold (Invitrogen, Carlsbad, CA, USA) and subsequently analyzed using the BioDocAnalyze (BDA) software (Biometra, Jena, Germany). Band position and volume (area intensity) were determined for all bands present using digital image analysis software (GelCompar II, Applied Maths, Sint-Martens-Latem, Belgium). The volume of each band was normalized to the total volume of all bands within each lane to determine the percent contribution of each band (bacterial phylotype) to the total species abundance within each sample. Multidimensional scaling was performed for optimized three-dimensional representation of the similarity matrix (GelCompar II, Applied Maths). Here, the Euclidian distances between two entries reflect the similarity between samples as well as possible. Discriminant analysis was performed by canonical analysis of principle coordinates (CAP) [5].

2. Results

2.1 PCR-DGGE

The most abundant *Ulva* species of the survey in the Ria Formosa, *U. rigida*, [6] was used along with *Fucus vesiculosus*, *Blidingia* sp. and *Z. noltii* to determine whether epibacterial communities from tidal pools differed from the surrounding bacterioplankton. To this end, 16SrRNA gene fragments PCR-amplified from epiphytic community metagenomic DNA were profiled by PCR-DGGE and compared with those obtained from water samples in the vicinity of the algae (Fig. S1). Thus, epiphytic and free-living bacteria were directly explored by PCR-DGGE without any bias towards cultivatable bacteria, providing a snapshot of bacterial community structuring in the surveyed samples. By means of multidimensional scaling analysis of the similarity matrix of PCR-DGGE band patterns, differences were observed between epibacterial communities on the macroalgae and free-living bacteria in their direct surroundings. The PCR-DGGE profiles retrieved from the seawater of tidal pools and the nearby main channel of the lagoon (Fig. S1A, W2-W8) showed high similarity and grouped closely in a principal coordinate analysis (PCO) (Fig. S1B). In contrast, the PCR-DGGE profiles of swabbed surface bacteria revealed a high diversity at the community level, even from the surfaces of *U. rigida* collected from the same environment (Fig. S1A, A10, A11), but were clearly separated from the water body profiles illustrated by the PCO analysis and confirmed by an additional supervised discriminant analysis (DA) with two groups (i) water samples (n = 7) and (ii) algal surface isolates (n = 6). The overall misclassification error of the cross-validation of the DA was only 8% indicating the significant differences between both groups.

We could not recognize PCR-DGGE bands from either free-living or algal surface communities that unequivocally matched the electrophoretic mobility of bands amplified from isolates retrieved from the surface of lab-cultured *U. mutabilis*. Therefore, to verify if morphogenesis-inducing bacteria could be isolated from their natural habitat (the Ria Formosa lagoon) and to determine whether they were phylogenetically similar to our MS2 and MS6 strains, we isolated, cultivated, and tested naturally occurring *Ulva* bacterial epibionts using the "*Ulva* bioassay array" for morphogenesis assessment (see main text).

S1 Fig. Bacterial fingerprinting based on the amplification of 16S rRNA. (A) DGGE shows the ribotypes of representative samples to survey the bacterial communities in seawater (W2-W8) and on algal surfaces (A10-A15). Lanes (S1, S9, and S15) represent a standard consisting of defined DNA of laboratory strains. Seawater samples have been taken from the following tidal pools (W2: pool #1; W3, 4, 5: pool #2; W6, 8: pool #3) and from a main channel of the lagoon (W7) in 2010. Bacterial analyzed were swabbed from the surface of *Ulva rigida* (A10: pool #1, A11: pool #2,), *Fucus vesiculosus* (A12: pool #2), *Blidingia* sp. (A13: pool #2, A14: pool #3) and from the seagrass *Zostera noltii* (A15) collected in a further tidal pool nearby. (**B**) The two origins of bacteria, seawater (turquoise circle) and algal/seagrass surface (black circle), were compared with each other based on the bacterial fingerprinting using unsupervised principal coordinate analysis (PCO) in order to visualize class distinctions. Axis 1 extracted 57% of the variation, axis 2 extracted 15% and axis 3 extracted 10%.

Figure S1

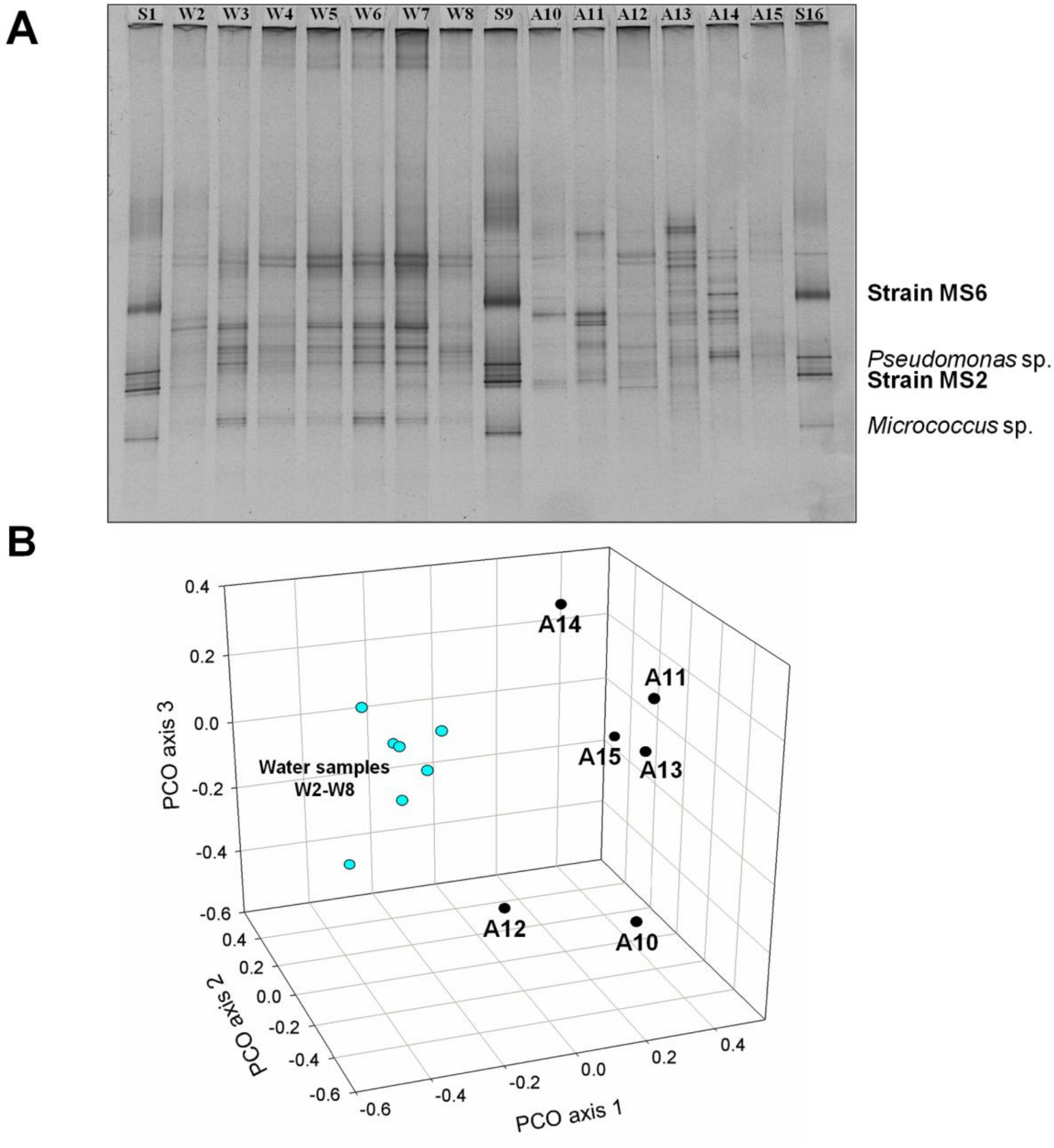
A
S1
W2
W3
W4
W5
W6
W7
W8
S9
A10
A11
A12
A13
A14
A15
S16
Strain MS6
Pseudomonas sp.
Strain MS2
Micrococcus sp.
B
PCO axis 3
PCO axis 2
PCO axis 1
0.4
0.2
0.0
-0.2
-0.4
-0.6
Water samples
W2-W8
A14
A11
A15
A13
A12
A10

3. References

1. Stratmann J, Paputsoglu G, Oertel W. Differentiation of *Ulva mutabilis* (Chlorophyta) gametangia and gamete release are controlled by extracellular inhibitors. J Phycol. 1996; 32: 1009-1021.
2. Muyzer G, Dewaal EC, Uitterlinden AG. Profiling of complex microbial populations by denaturing gradient gel electrophoresis analysis of polymerase chain reaction amplified genes coding for 16*S* ribosomal RNA. Appl Environ Microbiol. 1993; 59: 695-700.
3. Sanchez O, Gasol JM, Massana R, Mas J, Pedros-Alio C. Comparison of different denaturing gradient gel electrophoresis primer sets for the study of marine bacterioplankton communities. Appl Environ Microbiol. 2007; 73: 5962-5967.
4. Sneed JM, Pohnert G. The green macroalga Dictyosphaeria ocellata influences the structure of the bacterioplankton community through differential effects on individual bacterial phylotypes. FEMS Microbiol Ecol. 2011; 75: 242-254.
5. Anderson MJ, Robinson J. Generalised discriminant analysis based on distances. Aust Nz J Stat 2003; 45: 301-318.
6. Alsufyani T, Engelen AH, Diekmann OE, Kuegler S, Wichard T. Prevalence and mechanism of polyunsaturated aldehydes production in the green tide forming macroalgal genus *Ulva* (Ulvales, Chlorophyta). Chem Phys Lipids. 2014; 183: 100-109.
