# Supplementary figures and images for: Macroalgal Morphogenesis Induced by Waterborne Compounds and Bacteria in Coastal Seawater
Source: PLoS One. 2016 Jan 8;11(1):e0146307. doi: 10.1371/journal.pone.0146307 (PMC4720170; doi:10.1371/journal.pone.0146307)

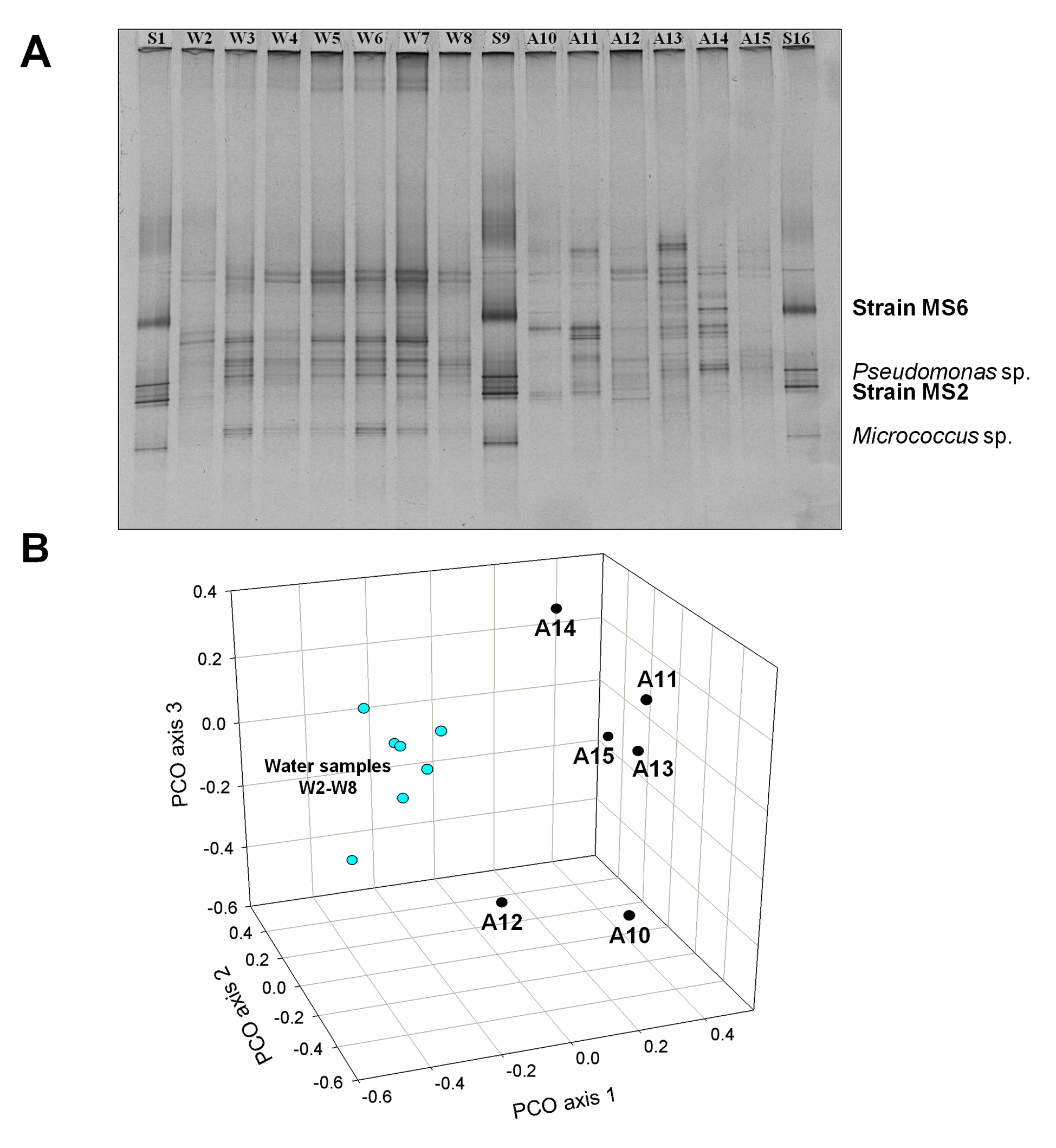

Supplement: S1 Fig — (A) DGGE shows the ribotypes of representative samples to survey the bacterial communities in seawater (W2-W8) and on algal surfaces (A10-A15). Lanes (S1, S9, and S15) represent a standard consisting of defined DNA of laboratory strains. Seawater samples have been taken from the following tidal pools (W2: pool #1; W3, 4, 5: pool #2; W6, 8: pool #3) and from a main channel of the lagoon (W7) in 2010. Bacterial analyzed were swabbed from the surface of Ulva rigida (A10: pool #1, A11: pool #2,), Fucus vesiculosus (A12: pool #2), Blidingia sp. (A13: pool #2, A14: pool #3) and from the seagrass Zostera noltii (A15) collected in a further tidal pool nearby. (B) The two origins of bacteria, seawater (turquoise circle) and algal/seagrass surface (black circle), were compared with each other based on the bacterial fingerprinting using unsupervised principal coordinate analysis (PCO) in order to visualize class distinctions. Axis 1 extracted 57% of the variation, axis 2 extracted 15% and axis 3 extracted 10%. (TIF) [file pone.0146307.s002.tif]
